# WD repeat-containing protein 1 maintains β-Catenin activity to promote pancreatic cancer aggressiveness

**DOI:** 10.1038/s41416-020-0929-0

**Published:** 2020-06-30

**Authors:** Hengchao Li, Xiaohui Liu, Shuheng Jiang, Xinwen Zhou, Lie Yao, Yang Di, Yongjian Jiang, Jichun Gu, Yishen Mao, Ji Li, Chen Jin, Pengyuan Yang, Deliang Fu

**Affiliations:** 1 Department of Pancreatic surgery, Huashan Hospital, Fudan University, Shanghai, 200040 P. R. China; 2 Institutes of Biomedical Sciences, Fudan University, Shanghai, 200032 P. R. China; 3 State Key Laboratory of Oncogenes and Related Genes, Shanghai Cancer Institute, Ren Ji Hospital, School of Medicine, Shanghai Jiao Tong University, Shanghai, 200240 P. R. China

**Keywords:** High-throughput screening, Paediatric cancer

## Abstract

**Background:**

The molecular signature underlying pancreatic ductal adenocarcinoma (PDAC) progression may include key proteins affecting the malignant phenotypes. Here, we aimed to identify the proteins implicated in PDAC with different tumour-node-metastasis (TNM) stages.

**Methods:**

Eight-plex isobaric tags coupled with two-dimensional liquid chromatography–tandem mass spectrometry were used to analyse the proteome of PDAC tissues with different TNM stages. A loss-of-function study was performed to evaluate the oncogenic roles of WD repeat-containing protein 1 (WDR1) in PDAC. The molecular mechanism by which WDR1 promotes PDAC progression was studied by real-time qPCR, Western blotting, proximity ligation assay and co-immunoprecipitation.

**Results:**

A total of 5036 proteins were identified, and 4708 proteins were quantified with high confidence. Compared with normal pancreatic tissues, 37 proteins were changed significantly in PDAC tissues of different stages. Moreover, 64 proteins were upregulated or downregulated in a stepwise manner as the TNM stages of PDAC increased, and 10 proteins were related to tumorigenesis. The functionally uncharacterised protein, WDR1, was highly expressed in PDAC and predicted a poor prognosis. WDR1 knockdown suppressed PDAC tumour growth and metastasis in vitro and in vivo. Moreover, WDR1 knockdown repressed the activity of the Wnt/β-Catenin pathway; ectopic expression of a stabilised form of β-Catenin restored the suppressive effects of WDR1 knockdown. Mechanistically, WDR1 interacted with USP7 to prevent ubiquitination-mediated degradation of β-Catenin.

**Conclusion:**

Our study identifies several previous functional unknown proteins implicated in the progression of PDAC, and provides new insight into the oncogenic roles of WDR1 in PDAC development.

## Background

Pancreatic ductal adenocarcinoma (PDAC) is the fourth leading cause of cancer-related deaths in industrialised countries, with an overall 5-year survival rate of only approximately 5%.^1^ In 2012, 43,920 new cases of PDAC were diagnosed, and 37,390 patients died of PDAC in the United States.^2^ Although early diagnosis and surgery and systemic chemotherapy for PDAC have greatly improved, the overall 5-year survival rate of PDAC patients is still poor.^3^ The tumour-node-metastasis (TNM) stage is an independent prognostic factor for the survival of PDAC patients.^4^ Thus, a better understanding of the molecular background underlying TNM stages may provide new prognostic biomarker candidates and offer new targets for the diagnosis, therapy and prognosis prediction for PDAC.^5–7^

Gene array and RNA sequencing, as two powerful tools for whole transcriptomic profile analysis, have become the preferred strategy to quickly identify differentially expressed genes in cancers.^8–10^ Accumulated RNA-sequencing studies have demonstrated the aberrant mRNA expression profiles of genes in PDAC.^11–13^ For example, Badea et al. analysed the expression of 36 PDAC tumours and matching normal pancreatic tissue samples using Affymetrix U133 Plus 2.0 whole-genome chips.^11^ However, little is known about proteomic changes during the malignant transformation of PDAC. Meanwhile, most expression-profiling studies have ignored the differences in patient clinical parameters, for example, the TNM stages of the cancer, which better predicts the survival discrimination and risk stratification. Isobaric tags for relative and absolute quantitation (iTRAQ) combined with two-dimensional liquid chromatography–tandem mass spectrometry (2DLC–MS/MS) are one of the most powerful methods in quantitative proteomics.^14,15^ iTRAQ can achieve sensitive and accurate protein quantification by utilising reporter ion pairs in the low mass range of MS2 spectra for quantitation. The multiplexing technique enables quantification of up to 8 different samples in a single MS-based experiment.^16^ Thus, it offers us a convenient method to simultaneously compare the proteomes of PDAC tissues.

To explore the dysregulated proteins implicated in the progression of PDAC, we performed iTRAQ labelling combined with 2DLC–MS/MS analysis in PDAC tissues and the corresponding paracancerous tissues from patients with different TNM stages (IIA, IIB, III and IV) and normal pancreas tissues. In total, 13 proteins related to PDAC progression were uncovered. Notably, the differentially expressed protein, WD repeat-containing protein 1 (WDR1), was identified by the proteomics approach and subsequently studied in detail.^17^ WDR1 participates in actin disassembly by severing actin filaments and dissociating actin monomers from filaments, and its significant roles in actin cytoskeletal remodelling have been demonstrated in the cytokinesis and cell migration of a variety of cells.^18–21^ In cancers, dysregulated WDR1 has been reported to regulate cell proliferation, migration and invasion.^22–24^ For example, highly expressed WDR1 promotes non-small-cell lung cancer cell proliferation and migration through actin cytoskeleton-mediated regulation of YAP.^22^ However, the oncogenic roles of WDR1 in PDAC are largely unknown.

The canonical Wnt/β-Catenin signalling pathway is evolutionarily conserved and plays important roles in many oncogenic processes, such as tumour initiation, progression, immune response and stemness maintenance.^25^ In the absence of Wnt signalling, β-Catenin is targeted by coordinated phosphorylation by CK1 and the APC/Axin/GSK-3β complex, resulting in ubiquitination-mediated degradation. In contrast, activation of the Wnt signalling leads to decreased activity of the APC/Axin/GSK-3β destruction complex. Stabilisation of β-Catenin is translocated to the nucleus and binds to LEF/TCF transcription factors to regulate Wnt target genes. USP7 is a ubiquitin- hydrolysing enzyme that is profoundly implicated in the diverse malignant phenotypes of cancers as it targets multiple substrates, such as TP53,^26^ N-MYC,^27^ Gli1^28^ and Yap.^29^ Recently, two reports have well documented how USP7 regulates β-Catenin ubiquitination and affects its stabilisation.

In this study, Western blotting and immunohistochemical analysis confirmed that the expression of WDR1 in PDAC tissues was upregulated in a stepwise manner as TNM stages increased. Subsequent loss-of-function studies showed that WDR1 contributes to PDAC tumour growth and metastasis. Mechanistically, we found that WDR1 can interact with USP7 to sustain β-Catenin activity by inhibiting ubiquitination-mediated degradation of β-Catenin.

## Methods

### Patients and tissue samples

The normal pancreatic head tissues (*n* = 5) were derived from the liver transplant donors without pancreatic disease; these samples remained after the donor liver was prepared. Surgically resected fresh tumour tissues and the corresponding paracancerous tissues were obtained from 20 PDAC patients who underwent initial pancreatic resection at the Huashan Hospital between June 2016 and December 2016. Fresh tissues were stored at −80 °C until use. A portion of tissues was preserved in 10% formalin solution for histopathological analysis. The tumour tissue samples were divided into four categories based on the TNM staging: stage IIA PDAC (*n* = 5), stage IIB PDAC (*n* = 5), stage III PDAC (*n* = 5) and stage IV PDAC (*n* = 5). The clinicopathological parameters of the PDAC patients enrolled in this study are listed in Supplementary Table 1. Eligibility criteria for selection of the subjects are shown in Supplementary Table 2. Tumours were classified according to the TNM staging system developed by the American Joint Committee on Cancer (AJCC, 7th edition)/The Union for International Cancer Control (UICC). The investigational protocol was approved by the Research Ethics Committee of Huashan Hospital, Fudan University, and all the patients were provided with written informed consent before enrolment. To determine the correlation between candidate proteins and TNM stages, we examined a PDAC tissue microarray, which contains 81 cases of PDAC and 44 non-tumour pancreas tissues (Shanghai Outdo Biotech Inc., Shanghai, China; OD-CT-DgPan01-006). Among the 81 patient samples, only 36 patients had follow-up data.

### Protein extraction and 8-plex iTRAQ labelling

The tumour and non-tumour pancreas tissue samples were homogenised and lysed in lysis buffer (7 M urea, 2 M thiourea, 1 mM phenylmethanesulfonylfluoride and protease inhibitor cocktails) at 4 °C for 1 h and centrifuged at 12,000 rpm for 30 min at 4 °C. The supernatant was collected, and the protein concentration was determined by the BCA Protein Assay Kit (Pierce Biotechnology, IL, USA). To diminish the effect of sample biological variation on the results of proteomics analysis, equal amounts of proteins from the tissue samples of five different individuals were pooled to generate one common sample for each type of tissue. Then, 120 μg of protein from each group was subjected to ice acetone precipitation overnight. After centrifugation, the pellet was resuspended in 20 μL of 500 mM triethylammonium bicarbonate (TEAB), and denaturant was added as a cosolvent. Subsequently, the resuspended proteins were reduced, alkylated and digested with trypsin according to the manufacturer’s protocol (Applied Biosystems, Framingham, MA, USA). Samples were iTRAQ-labelled as follows: normal pancreas tissues, 113; stage IIA tumour tissues, 114; stage IIB tumour tissues, 115; mixed stage IIA and IIB paracancerous tissues, 116; stage III paracancerous tissues, 117; stage III tumour tissues, 118; stage IV paracancerous tissues, 119; stage IV tumour tissues, 121. The labelled peptide samples were then pooled and lyophilised in a vacuum concentrator prior to SCX fractionation. All the samples were detected three times.

### LC–MS/MS analysis

The peptide mixtures were resuspended in buffer A (5 mM ammonium formate containing 2% acetonitrile, pH 10) and fractioned using a high-pH RPLC column (Waters, Xbridge C18 3.5 µm, 150 × 2.1 mm) at a constant flow rate of 0.2 mL/min. The gradient elution was performed by 0–35% B (5–45 min) on a UPLC system (Waters, USA). The wavelength of the UV detector was set at 280 nm. A total of 20 fractions were collected and then mixed into 10 fractions, which were dried in a vacuum concentrator for subsequent nano-LC–MS/MS analysis. The LC–MS/MS analysis was performed in triplicate. Each fraction was analysed on an Easy-nLC 1000 system (Thermo Scientific, MA, USA) coupled with an Orbitrap Fusion Tribrid MS platform (Thermo Scientific, MA, USA) with a 25-cm-long column (75 μm id × 25-cm long, packed with 2 μm id, 100-Å pore size, C18 packing material, Thermo Scientific, MA, USA). Peptides were eluted in 120 min with a gradient of mobile phase B (acetonitrile and 0.1% formic acid) from 5 to 7% (0–5 min), 7 to 23% B (5–70 min) and 23 to 45% B (70–105 min) at a flow rate of 300 nL/min. The Obitrap Fusion analysis was performed in a data-dependent acquisition mode. The MS1 scans over a mass range of m/z 350–1500 with detection in the Obitrap (120-K resolution), and the autogain control (AGC) was set to 1 × 10^5^. Survey full-scan MS spectra (m/z 300–1200) were acquired with a mass resolution of 70 K. The most intense ions above the threshold ion count of 30,000 in MS1 were selected for sequential high-energy collisional dissociation (HCD) MS/MS scans with a resolution of 17.5 K. All data were acquired with Xcalibur software v3.0.63 (Tune v2.0 1258).

### Data accessibility

The raw MS data are available at iPROXY. The link is http://www.iprox.org/page/PSV023.html;?url=1517189976490aI9r and the password is 58KZ.

### Data analysis and functional interpretation

Protein identification and quantification for iTRAQ experiments were carried out using Proteome Discovery version 1.3 (PD, Thermo Scientific, MA, USA) using MASCOT search engine. A database search was performed against a target-decoy database constructed based on a SwissProt human database (84,433 entries). Only proteins with a false discovery rate (FDR) < 1% were reported. iTRAQ reporter ion intensities of these peptides were referenced to obtain relative quantifications of proteins. Proteins with two peptides identified were considered for further analysis. In addition, IPA software (http://www.ingenuity.com) was used for complete functional, pathway and network analysis.

### Gene ontology and bioinformatics analysis

Differentially expressed proteins were annotated by Gene Ontology (GO) using DAVID software (version 6.7). The GO terms with computed P values less than 0.05 were considered significantly enriched. Ingenuity Pathway Analysis Tool (IPA, Ingenuity^®^ Systems, Redwood City, CA) was used to analyse and visualise the data to reveal molecular interactions and canonical pathways during the pancreatic tumour process. The RNA-sequencing data for PDAC used in this study were downloaded from The Cancer Genome Atlas (TCGA) database (https://cancergenome.nih.gov/). The mRNA expression data containing log_2_-transformed RNA-seq by expectation maximisation (RSEM) values were summarised at the gene level.

### Cell culture and reagents

Human PDAC cells were obtained from the Type Culture Collection of the Chinese Academy of Sciences (Shanghai, China). MiaPaca2 cells were obtained from American Type Culture Collection (ATCC). PANC28 cell line is a gift from Dr. Yanguang Chen. All the cells were grown at 37 °C in a humidified atmosphere of 95% air and 5% CO_2_, using Dulbecco’s Modified Eagle Medium (DMEM) or RPMI-1640 medium with 10% FBS. Cycloheximide (CHX) was purchased from Sigma-Aldrich (Shanghai, Chian). HBX19818 was obtained from MedChemExpress (HY17540, Shanghai, China). The specific USP7 and WDR1 shRNA plasmids, β-Catenin S33Y plasmid and pcDNA3.1-USP7 plasmid were designed and synthesised by Genepharma (Shanghai, China).

### Western blotting

Western blotting analysis was performed on all individual samples in the identification cohort. For sodium dodecyl sulfate polyacrylamide gel electrophoresis (SDS-PAGE), 20 μg of protein was separated by gel electrophoresis on 10% Mini-PROTEAN TGX Precast Gels (#456-1084; Bio-Rad Laboratories, Shanghai, China) and transferred to polyvinylidene fluoride membranes. The membranes were then incubated at 4°C overnight with primary anti-WDR1 antibody (1:1000, ab228738, Abcam, Shanghai, China), primary anti-USP7 antibody (1:1000, ab4080, Abcam, Shanghai, China) or primary anti-β-Catenin antibody (1:5000, ab32572, Abcam, Shanghai, China), followed by incubation with a horseradish peroxidase-conjugated secondary antibody (1:1000, GE Healthcare Biosciences, NY, USA) for 1 h at room temperature. β-actin was used as a control for protein loading, and was detected using a mouse monoclonal anti-β-actin antibody (1:1000, ab5694, Abcam, Shanghai, China). The signal was visualised using an enhanced chemiluminescence agent (GE Healthcare Biosciences, NY, USA), and quantitative analysis of the protein bands was performed by using ImageJ software (NIH Image).

### Co-immunoprecipitation (Co-IP)

Co-IP was performed as described previously.^30^ In brief, PDAC cells were collected and lysed with IP lysis buffer (Beyotime, Shanghai, China). Total protein was incubated with 50 μl of Protein G-agarose suspension (Millipore, USA). After supplementation for 2 h at 4 °C, the beads were removed, and the supernatant was incubated with the primary antibodies for an additional 2 h at 4°C. Then, 100 μl of Protein G-agarose was added. After incubation at 4 °C overnight, the immunoprecipitates were collected and washed three times with 1×PBS. Finally, the loading buffer was added, and the agarose was boiled and subjected to Western blotting analysis.

### Immunohistochemistry

Immunohistochemical staining analysis for WDR1 was performed on formalin-fixed, paraffin-embedded tissue sections as reported previously.^30^ Briefly, the sections were deparaffinised, rehydrated, blocked with H_2_O_2_, washed with PBS three times and then incubated with anti-WDR1 antibody (1:200, ab228738, Abcam, Shanghai, China) overnight at 4 °C. The next day, the slides were washed, incubated with horseradish peroxidase-conjugated secondary antibody and developed with diaminobenzidine (DAB) solution. Then, the slides were counterstained with haematoxylin and examined under an optical microscope (Carl Zeiss, Germany). Scoring standards included the intensity of staining, scored as 0 (negative), 1 (weak), 2 (moderate) and 3 (strong), and the percentage of positive cells, scored as 0 (no staining), 1 (<25%), 2 (25–50%), 3 (50–75%) and 4 (>75%). The product of the score of the staining intensity and the score of staining extent constituted the total score. We defined positive cases as a score of >2.

### Plate colony-formation assay

The plate colony-formation experiment was used to determine the proliferation potential of PDAC cells. In brief, PDAC cells were seeded at 500 cells in 2 ml of DMEM or RPMI-1640 medium per well in six-well culture plates. After culture for 10–12 days, cells were fixed with 4% paraformaldehyde and stained with crystal violet.

### Cell invasion assay

Boyden chambers with filter inserts (8 μm) coated with Matrigel in 24-well tissue culture plates were used for cell-invasive potential analysis. Briefly, 2 × 10^5^ cells in 100 μl of serum-free medium were placed in the upper chamber, and 0.7 ml of medium containing 10% FBS was placed in the lower chamber. After 48 h in culture, cells were fixed with 4% paraformaldehyde and stained with crystal violet. Cells on the upper side of the filters were removed with cotton-tipped swabs. Migrated cells on the underside of the filters were d counted under a fluorescence microscope.

### Animal experiments

The athymic male nu/nu mice were used for animal experiments in this study. Mice were housed and reared in specific pathogen-free and barrier conditions. All animals were housed in a controlled environment under a 12-h dark/light cycle with free access to food and water. For the subcutaneous xenograft experiment, a total of 1 × 10^6^ sh-Ctrl or sh-WDR1 PANC1 cells were injected subcutaneously into the lower back. Tumour volumes were monitored every week. Tumour volumes were calculated as length × width^2^/2. Six weeks later, the mice were sacrificed, and the tumour tissues were isolated, and the tumour weight was determined. For lung metastasis experiments, a total of 1 × 10^6^ sh-Ctrl or sh-WDR1 PANC1 cells in 100 μl of Hank’s buffered saline were intravenously injected, and lung colonisation was quantified after 4 weeks. Specifically, sevoflurane was used for mouse anaesthesia. At protocol-defined endpoints, mice were killed by carbon dioxide inhalation. All animals received humane care according to the criteria outlined in the “Guide for the Care and Use of Laboratory Animals” prepared by the National Academy of Sciences and published by the National Institutes of Health (NIH publication 86-23, revised 1985). All manipulations were approved by the Research Ethics Committee of Huashan Hospital, Fudan University.

### RNA-sequencing experiment

The total RNA from sh-Ctrl and sh-WDR1 PANC1 cells was isolated using TRIzol reagent for RNA sequencing following the manufacturer’s instructions. The RNA quality was checked using an Agilent 2100 Bioanalyzer (Agilent Technology, USA). The library fragments were purified with THE AMPure XP system (Beckman Coulter, Beverly, USA). The clustering of the index-coded samples was performed on a cBot Cluster Generation System using TruSeq PE Cluster Kit v3-cBot-HS (Illumina) according to the manufacturer’s instructions. After cluster generation, the library preparations were sequenced on an Illumina HiSeq X Ten and 150-bp paired-end reads were generated. HTSeq v0.6.0 was used to count the reads numbers mapped to each gene. Then FPKM of each gene was calculated based on the length of the gene and read count mapped to this gene.

### Real-time quantitative PCR analysis

Total RNA was isolated from PDAC cells using TRIzol reagent. cDNA preparation was performed according to standard procedures using a primeScript RT Master kit (Takara, Japan). The SYBR green-based real-time quantitative transcription–polymerase chain reaction assay was used to determine the mRNA level of the indicated genes using the ViiA7 System (Applied Biosystems, Foster City, CA). ACTB served as a control for RNA quantity. The primers for mRNA-level detection were as follows: WDR1 forward, 5′- CGGGTACATCAACTATCTGGACA-3′, WDR1 reverse, 5′-GTTTTTATGCACCGTCAGACAC-3′; USP7 forward, 5′-GGAAGCGGGAGATACAGATGA-3′, USP7 reverse, 5′-AAGGACCGACTCACTCAGTCT-3′; c-Myc forward, 5′-GGCTCCTGGCAAAAGGTCA-3′, c-Myc reverse, 5′-CTGCGTAGTTGTGCTGATGT-3′; MMP9 forward, 5′-TGTACCGCTATGGTTACACTCG-3′, MMP9 reverse, 5′-GGCAGGGACAGTTGCTTCT-3′; CCND1 forward, 5′-CAATGACCCCGCACGATTTC-3′, CCND1 reverse, 5′-CATGGAGGGCGGATTGGAA-3’; ACTB forward, 5′-CATGTACGTTGCTATCCAGGC-3′, ACTB reverse, 5′-CTCCTTAATGTCACGCACGAT-3′. Relative mRNA expression was calculated using the 2^(−ΔΔCt)^ method and normalised to ACTB mRNA levels.

### Luciferase reporter assay

The TOPFlash luciferase assay was used to determine the activity of Wnt/β-Catenin signalling. In brief, PDAC cells in a 96-well plate were transfected with 100 ng of the reporter plasmid TOP or FOP and 10 ng of TK/Renilla for each well. After 36 h of transfection, cells were lysed with passive lysis buffer, and the luciferase activity was measured according to the manufacturer’s instructions (Promega, USA).

### In situ proximity ligation assay (PLA)

PLA experiment was performed as described previously.^30^ In brief, PDAC cells were fixed with 4% formaldehyde for 30 min at room temperature, followed by permeabilisation with 0.05% Triton X-100 for 5 min. After incubation with the blocking buffer, PANC1 and AsPC1 cells were incubated with primary antibodies against WDR1 and USP7 from two different species at 4 °C overnight. The next day, the cells were washed three times with wash buffer and incubated with species-specific PLA probes in a preheated humidity chamber for 1 h at 37 °C. Then, the ligation solution, consisting of two additional oligonucleotides and ligase, was added to join the hybridised oligonucleotides to form a closed circle. Finally, cells were counterstained with DAPI to locate the nuclei and subjected to signal detection.

### Statistical analysis

Statistical analyses were performed using GraphPad Prism software version 5.0 (GraphPad Software, Inc., La Jolla, CA, USA). Student’s *t* test or one-way ANOVA was used to determine significant differences between different groups. The χ^2^ test was used to determine the different groups in immunohistochemical staining analysis. Cumulative survival time was calculated using the Kaplan–Meier method and analysed by the log-rank test. Correlation analysis in this study was determined using Spearman’s test. *P* values < 0.05 were considered statistically significant.

## Results

### Identification of proteins implicated in PDAC progression

**Fig. 1:**
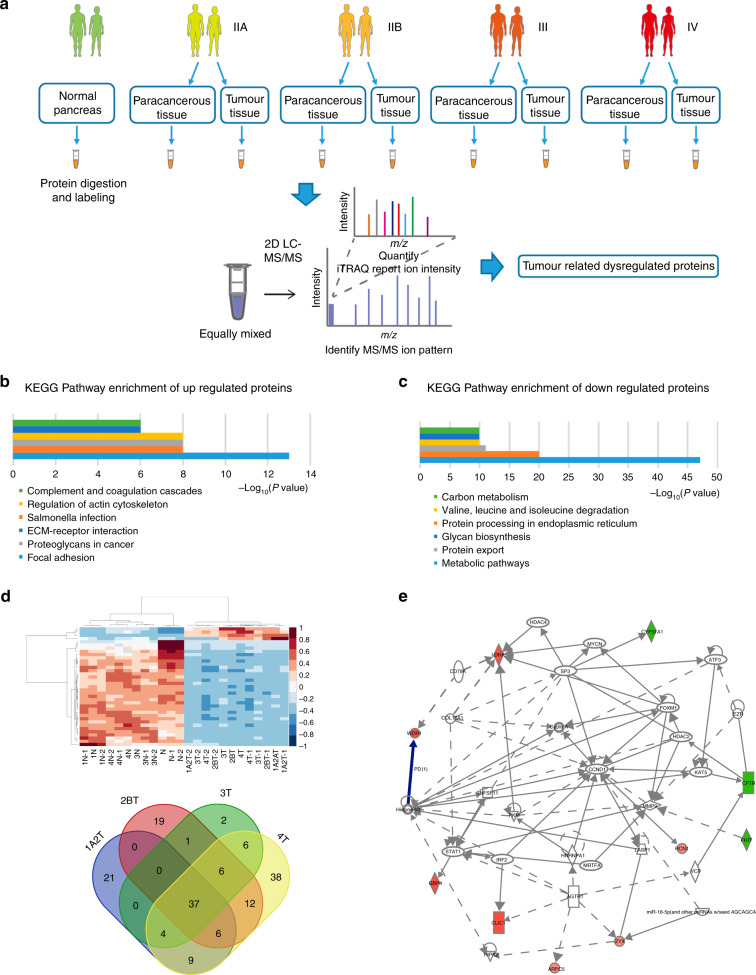
The differentially expressed proteins identified by iTRAQ–2DLC–MS/MS. **a** Flowchart of the experimental set-up. Fresh PDAC and the corresponding normal tissue samples were harvested from newly diagnosed PDAC patients. Samples were pooled and subsequently identified by mass spectrometry, and data were then normalised for subsequent data analysis. **b** Gene ontology analysis of differentially expressed proteins with fold change ≥1.2 and *P* value ≤ 0.05. **c** Gene ontology analysis of differentially expressed proteins with fold change < 1.2 and *P* value ≤ 0.05. **d** Heatmap and Venn diagram showing the 37 upregulated or downregulated proteins across all stages of PDAC tissues. **e** IPA analysis showed that the 10 differentially expressed proteins were involved in tumorigenesis. Selected upregulated proteins are marked in red and downregulated proteins are marked in green.

Using iTRAQ–2DLC–MS/MS, a comparative study was performed to analyse the dynamic changes in protein expression throughout the different TNM stages (7th edition AJCC/UICC Classification of Malignant Tumors) (Fig. 1a). As a result, a total of 5036 proteins were identified with FDR < 0.1%, with 4893, 4907 and 4905 proteins quantified in the three independent experiments. Among these proteins, 4708 proteins were identified and quantified with two peptides detected with high confidence, so they were analysed further. Detailed information of identification and quantification was provided in Supplementary Table 3. Comparing the protein expression level in the cancer tissue with that in the non-tumour tissue, 623 proteins were found to have significant differences with |fold change | ≥1.2 and *P* value ≤ 0.05 (Supplementary Table 4). Gene ontology analysis showed that the upregulated proteins were profoundly implicated in focal adhesion, salmonella infection, proteoglycans in cancer, regulation of the cytoskeleton, ECM–receptor interaction and complement and coagulation cascades (Fig. 1b), while the downregulated proteins were critically involved in metabolic pathways, protein processing in endoplasmic reticulum, protein export, valine, leucine and isoleucine degradation, N-glycan biosynthesis and carbon metabolism (Fig. 1c). Notably, 37 proteins were upregulated or downregulated in all stages of PDAC tissues (Fig. 1d; Supplementary Table 5).

Moreover, 64 proteins were found to be gradually upregulated or downregulated in PDAC tissues of different stages compared with normal pancreas tissues (Supplementary Table 6). For in-depth analysis of the interactions between these candidate proteins, a subnetwork with the shortest path to describe the interaction of each protein was constructed based on IPA analysis. As shown in Fig. 1e and Table 1, interaction network analysis showed that ten differentially expressed proteins were involved in tumorigenesis, suggesting that the dysregulation of these proteins might contribute to the tumour progression of PDAC.

**Table 1:** A list of differentially expressed proteins (*n* = 10) identified by iTRAQ labelling combined with 2DLC–MS/MS.

No.	Accession	Protein	Average value				
			N	IIA	IIB	III	IV
1	LDHA	Lactate dehydrogenase A	1.29	1.59	1.79	2.84	2.72
2	WDR1	WD repeat-containing protein 1	1.05	1.31	1.35	1.43	1.53
3	GBP4	Guanylate-binding protein 4	1.07	1.55	1.54	1.44	1.44
4	CLIC1	Chloride intracellular channel 1	1.21	1.60	1.76	1.87	1.93
5	ARPC5	Actin-related protein 2/3 complex subunit 5	0.93	1.27	1.29	1.37	1.43
6	ZYX	Zyxin	1.05	1.35	1.36	1.38	1.49
7	RCN1	Reticulocalbin 1	1.04	1.27	1.42	1.49	1.61
8	FHIT	Fragile histidine triad diadenosine triphosphatase	0.91	0.69	0.66	0.63	0.56
9	CFTR	CF transmembrane conductance regulator	0.91	0.62	0.61	0.59	0.57
10	CYP51A1	Cytochrome P450 Family 51 Subfamily A Member 1	0.97	0.79	0.76	0.71	0.67

### Validation of the differentially expressed protein WDR1

**Fig. 2:**
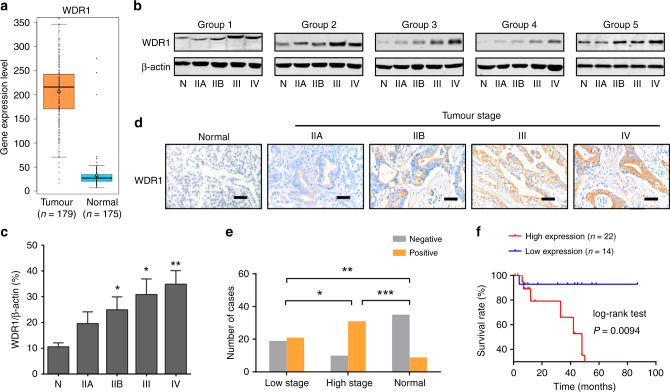
The expression pattern of WDR1 in pancreatic cancer. **a** Combined data from TCGA dataset and GTEx portal (http://gemini.cancer-pku.cn/) showed that the mRNA expression level of *WDR1* in pancreatic cancer (*n* = 179) was significantly higher than that in normal pancreas tissues (*n* = 175). **b** Western blotting shows the protein expression levels of WDR1 in normal pancreas and pancreatic cancer with different TNM stages. **c** Quantitative analysis of the expression levels (relative band density) of WDR1 in normal pancreas (N), and pancreatic cancer with different TNM stages (IIA, IIB, III and IV). **d** Representative images of WDR1 staining in normal pancreas and PDAC tissues with increasing TNM stages. **e** The frequency distribution of WDR1 expression in low-stage PDAC tissues (*n* = 40), high-stage PDAC tissues (*n* = 41) and normal pancreas tissues (*n* = 44). **f** Kaplan–Meier analysis of the overall survival of PDAC patients based on the protein expression of WDR1. *P < 0.05, **P < 0.01, ***P < 0.001.

Among the differentially expressed proteins identified by proteomic analysis, the typically upregulated protein WDR1, which has not been functionally studied in PDAC, was selected for further characterisation. By data mining the TCGA cohort and GTEx portal, we found that the mRNA expression level of *WDR1* in pancreatic cancer (*n* = 179) was significantly higher than that in normal pancreas tissues (*n* = 175), suggesting the overexpression profiles of WDR1 in PDAC (Fig. 2a). Then, we measured the protein expression of WDR1 in all individual samples in the identification cohort by Western blotting (Fig. 2b). Consistent with the observation from MS analysis, the results showed that WDR1 protein levels were increased from normal pancreas tissues to PDAC with different TNM stages in a stepwise manner (Fig. 2c). Furthermore, immunohistochemistry was performed to validate the expression pattern of WDR1 in the identification cohort. As a result, the cytoplasmic immunoreactivity of WDR1 can be merely detected in non-tumour tissues, but gradually upregulated in PDAC as TNM stage increased (Fig. 2d). Finally, by analysis of an independent cohort of PDAC tissue microarrays containing 81 PDAC and 44 adjacent non-tumour tissues, we showed that there was a significant difference in the protein levels of WDR1 in all the pairwise comparisons of the three groups (PDAC tissues with low TNM stage, PDAC tissues with high TNM stage and adjacent non-tumour tissues) (Fig. 2e). Kaplan–Meier curve analysis showed that PDAC patients with higher WDR1 protein expression had a poor prognosis (Fig. 2f). Collectively, these data suggest that WDR1 might act as a candidate biomarker for the progression of PDAC.

### WDR1 knockdown inhibits tumour growth and metastasis in PDAC

**Fig. 3:**
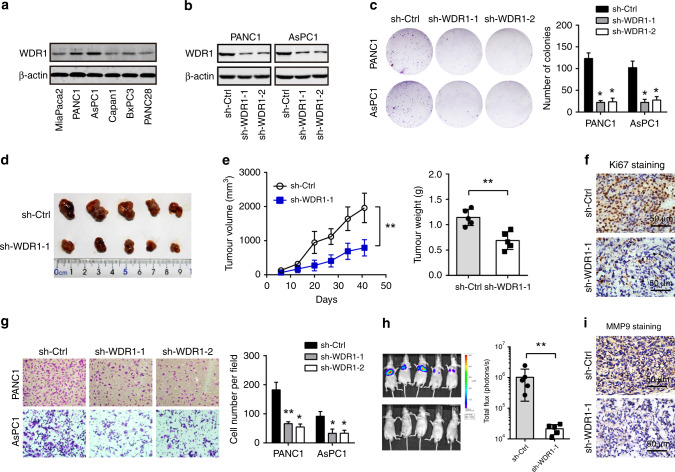
WDR1 knockdown suppresses tumour growth and metastasis of pancreatic cancer. **a** Western blotting analysis of WDR1 protein expression in PDAC cells. **b** The shRNA-mediated WDR1-knockdown efficiency in PANC1 and AsPC1 cells was analysed by Western blotting. **c** The effect of WDR1 knockdown on in vitro PDAC cell proliferation was determined by plate colony-formation assay. **d** The effect of WDR1 knockdown on in vivo PDAC tumour growth was studied by PANC1-derived subcutaneous xenograft model. **e** The tumour volume and weight of sh-Ctrl and sh-WDR1 PANC1-derived subcutaneous xenograft tumour tissues. **f** Representative immunohistochemical images of Ki67 staining from sh-Ctrl and sh-WDR1 PANC1-derived tumour tissues. **g** The effect of WDR1 knockdown on in vitro PDAC cell invasion was determined by transwell assay. **h** The effect of WDR1 knockdown on in vivo PDAC tumour metastasis; the sh-Ctrl and sh-WDR1 PANC1 cells were injected into the tail vein of BALB/c nude mice. After 30 days, lung metastasis was measured by an in vivo imaging system. **i** Representative immunohistochemical images of MMP9 staining from sh-Ctrl and sh-WDR1 lung metastasis tumour tissues. *P < 0.05, **P < 0.01.

To determine the potential biological functions of WDR1 in PDAC, we carried out loss-of-function studies in PDAC cell lines. Endogenous WDR1 in PDAC cells was detected by Western blotting (Fig. 3a). Two cell lines, PANC1 and AsPC1, with higher WDR1 expression, were subjected to knockdown experiments. Two specific shRNAs targeting WDR1 resulted in a significant reduction in WDR1 protein levels (Fig. 3b). The plate colony-formation assay showed that WDR1 knockdown led to pronounced inhibition of cell proliferation (Fig. 3c). By using subcutaneous xenograft model, we found that WDR1 knockdown suppressed tumour growth as revealed by changes in tumour volume and weight (Fig. 3d–e). IHC analysis showed that tumour cells with positive staining of Ki67 in the sh-WDR1 group were markedly attenuated compared with those in the sh-Ctrl group (Fig. 3f). Furthermore, transwell experiments showed that WDR1 knockdown inhibited the cell-invasive capacity of PANC1 and AsPC1 cells (Fig. 3g). Consistently, WDR1 knockdown also remarkably reduced the in vivo lung metastasis of PDAC cells (Fig. 3h). IHC analysis showed that immunoreactivity of the invasive marker MMP9 was drastically downregulated in sh-WDR1 tumour tissues (Fig. 3i). Taken together, these findings suggest that WDR1 is profoundly implicated in the malignant phenotypes of PDAC.

### WDR1 correlates with Wnt/β-Catenin signalling activity in pancreatic cancer

**Fig. 4:**
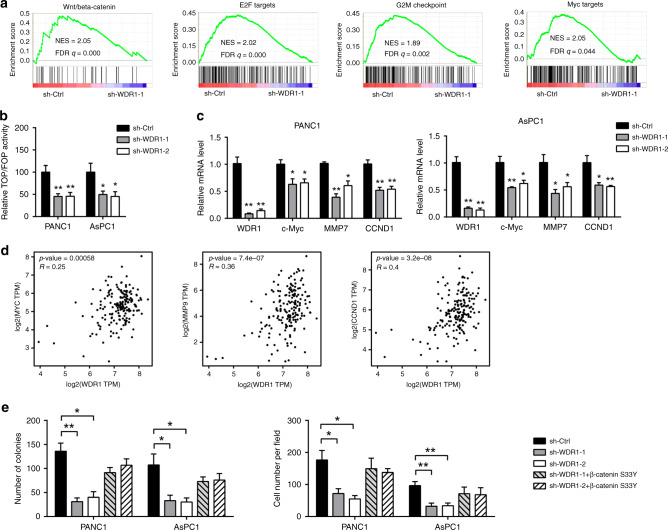
WDR1 correlates with Wnt/β-Catenin signalling activity in pancreatic cancer. **a** Gene set enrichment analysis of RNA-seq data of sh-Ctrl and sh-WDR1 PANC1 cells. **b** Relative TOP/FOP activities of sh-Ctrl and sh-WDR1 PANC1 and AsPC1 cells. **c** Real-time qPCR analysis of the effect of WDR1 knockdown on the expression of downstream targets of Wnt/β-Catenin signalling. **d** Correlation analysis of WDR1 with c-Myc, MMP9 and CCND1 in the TCGA PAAD cohort. **e** The effect of WDR1 knockdown on in vitro PDAC cell proliferation and invasion upon transfection of β-Catenin S33Y mutant plasmids. *P < 0.05, **P < 0.01.

To uncover the mechanism by which WDR1 facilitates tumour growth and metastasis, RNA-sequencing analysis of sh-Ctrl and sh-WDR1–1 PANC1 cells was used to determine transcriptional changes. Gene set enrichment analysis (GSEA) showed that WDR1 knockdown led to decreased enrichment of Wnt/β-Catenin activity, E2F targets, G2M checkpoint and Myc targets (Fig. 4a). Given the critical roles of the Wnt/β-Catenin signalling pathway in PDAC progression, we hypothesised that WDR1 may affect PDAC development by modulating Wnt/β-Catenin signalling. To test this hypothesis, we first measured TOP/FOPFlash luciferase activity after WDR1 knockdown. As a result, a dramatic inhibition in Wnt/TCF transcription was observed in sh-WDR1 PDAC cells (Fig. 4b). By using real-time qPCR analysis, we noticed that the expression of the downstream targets of Wnt/β-Catenin signalling, including c-Myc, MMP7 and CCND1, was significantly downregulated by genetic silencing of WDR1 in PANC1 and AsPC1 cells (Fig. 4c). Data from the TCGA cohort showed that there was a close correlation between WDR1 and MYC, MMP9 and CCND1 in PDAC (Fig. 4d). Moreover, ectopic expression of a stabilised form of β-Catenin (β-Catenin Ser33Y) largely rescued the inhibitory effect of WDR1 knockdown on cell proliferation and invasion of PANC1 and AsPC1 cells (Fig. 4e). Thus, these results strongly support that WDR1 is a regulator of Wnt/β-Catenin signalling in PDAC.

### WDR1 interacts with USP7 to regulate β-Catenin ubiquitination in pancreatic cancer

**Fig. 5:**
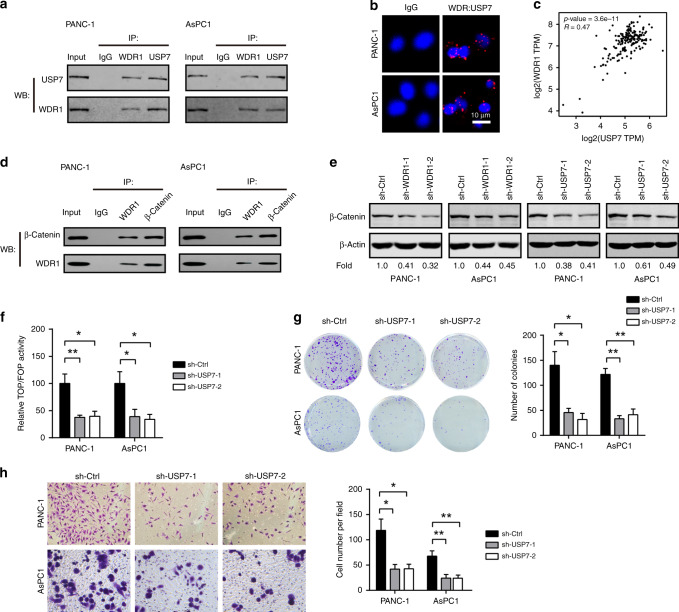
WDR1 interacts with USP7 and β-Catenin in pancreatic cancer. **a** Co-immunoprecipitation (Co-IP) analysis of the interaction between WDR1 and USP7 in PANC1 and AsPC1 cells. **b** In situ proximity ligation assay (PLA) analysis of the interaction between WDR1 and USP7 in PANC1 and AsPC1 cells. **c** Correlation analysis of WDR1 with USP7 in the TCGA PAAD cohort. **d** Co-IP analysis of the interaction between WDR1 and β-Catenin in PANC1 and AsPC1 cells. **e** Western blotting analysis of the effects of WDR1 or USP7 on β-Catenin expression in PANC1 and AsPC1 cells. **f** Relative TOP/FOP activities of sh-Ctrl and sh-USP7 PANC1 and AsPC1 cells. **g** The effect of USP7 knockdown on in vitro PDAC cell proliferation was determined by plate colony- formation assay. **h** The effect of USP7 knockdown on in vitro PDAC cell invasion was determined by transwell assay. *P < 0.05, **P < 0.01.

The conserved Wnt/β-Catenin pathway plays versatile roles during tumour initiation and development.^25,31^ The key component of the pathway, β-catenin, is tightly regulated by phosphorylation and ubiquitination-mediated degradation.^32^ Of note, protein ubiquitination-mediated degradation is a reversible process modulated by E3 ubiquitin ligases and deubiquitinating enzymes. By data mining the BioGRID database (https://thebiogrid.org/), we found that many proteins involved in ubiquitination-mediated degradation are predicted to interact with WDR1, especially the deubiquitinating enzyme USP7. By co-immunoprecipitation (Co-IP) assay, we found that WDR1 can physically interact with USP7 in PANC1 and AsPC1 cells (Fig. 5a). Using proximity ligation assay, we further confirmed the interaction between WDR1 and USP7 in PDAC cells (Fig. 5b). In addition, a close correlation between WDR1 and USP7 in the TCGA PDAC cohort was found (Fig. 5c). Co-immunoprecipitation experiment also revealed the physical interaction between WDR1 and β-Catenin in PANC1 and AsPC1 cells (Fig. 5d). Western blotting showed that either WDR1 or USP7 knockdown downregulated β-Catenin in PDAC cells (Fig. 5e). Moreover, USP7 knockdown in PDAC cells also suppressed TOPFlash luciferase activity (Fig. 5f), cell proliferation activity (Fig. 5g) and invasive potential (Fig. 5h). However, ectopic expression of USP7 failed to restore the inhibitory effects of WDR1 knockdown on cell proliferation and invasion (Supplementary Fig. 1).

**Fig. 6:**
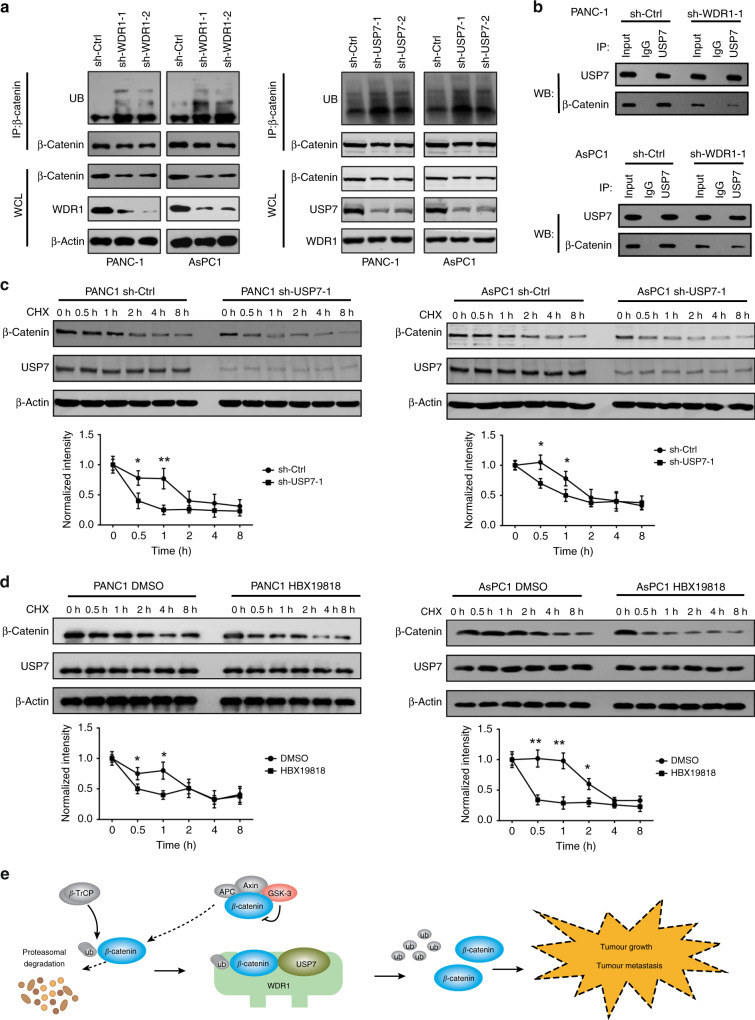
WDR1 and USP7 regulate β-Catenin ubiquitination in pancreatic cancer. **a** Cell lysates from sh-Ctrl and sh-USP7 PANC1 and AsPC1 cells were immunoprecipitated with anti-β-Catenin antibody, and the immunocomplexes were immunoblotted with antibodies against UB and β-Catenin. **b** Co-IP analysis of the interaction between USP7 and β-Catenin in sh-Ctrl and sh-WDR1–1 PANC1 and AsPC1 cells. **c** The sh-Ctrl and sh-USP7 PANC1 and AsPC1 cells were treated with 100 μg/ml CHX for the indicated times; then, the cell extracts were harvested, and subjected to immunoblotting with the indicated antibodies. **d** In the presence or absence of HBX19818, PANC1 and AsPC1 cells were treated with 100 μg/ml CHX for the indicated times; then, the cell extracts were harvested and subjected to immunoblotting with the indicated antibodies. **e** A schematic diagram shows the mechanism by which WDR1 interacts with USP7 to promote β-Catenin deubiquitination in pancreatic cancer. WDR1 might act as a scaffold to bridges Usp7 and β-Catenin and enhance USP7-mediated β-Catenin deubiquitination.

To uncover the mechanism by which WDR1 and USP7 regulate β-Catenin ubiquitination in PDAC cells, we first determined the levels of polyubiquitin-modified β-Catenin in PANC1 and AsPC1 cells upon WDR1 or USP7 knockdown. As shown in Fig. 6a, WDR1 or USP7 knockdown markedly increased the levels of β-Catenin ubiquitination. WDR1 knockdown did not affect the mRNA or protein level of USP7 (Supplementary Fig. 2). However, WDR1 knockdown significantly reduced the interaction between β-Catenin and USP7 in PANC1 and AsPC1 cells (Fig. 6b). To investigate whether USP7 stabilises β-Catenin protein, we measured the implication of USP7 knockdown or inhibition on the stability of endogenous β-Catenin protein in the presence of 100 μg/ml cycloheximide (CHX, an inhibitor of protein translation). The result showed that β-Catenin protein was degraded more rapidly in sh-USP7 cells than in sh-Ctrl cells (Fig. 6c). Similarly, inhibition of USP7 by HBX19818 promoted β-Catenin degradation in PANC1 and AsPC1 cells (Fig. 6d). Collectively, these data indicate that WDR1 and USP7 are involved in β-Catenin deubiquitination in PDAC cells (Fig. 6e).

## Discussion

In this study, we successfully acquired a comprehensive and detailed proteome expression profile involved in the progression of PDAC by using iTRAQ–2DLC–MS/MS technology. Altogether, 5036 nonredundant proteins were confidently quantified, 82 proteins were found to be gradually upregulated and 186 were found to be continuously downregulated along with TNM stages. Among them, the functional unidentified protein, WDR1, was validated by using Western blotting and immunohistochemistry. Further functional and mechanistic analysis suggested its potential oncogenic roles in PDAC.

To the best of our knowledge, this is the first study to establish a correlation of the whole protein expression profiles with PDAC at different TNM stages in the Chinese population, providing a preliminary study of the tumour stage-specific proteins in PDAC. The majority of the quantified proteins acquired in this study have been demonstrated in PDAC, and our results further confirm their essential roles in the progression of PDAC. Importantly, many other proteins that were previously unreported in PDAC, such as WDR1, were also revealed in this study. Our data suggested that these differentially expressed proteins might be involved in PDAC development.

WDR1, also known as actin-interacting protein 1 (AIP1), is a highly conserved protein containing 9 WD repeats, and is ubiquitously expressed in eukaryotes. WDR1 promotes cofilin-mediated actin filament disassembly, and plays a crucial role in cytokinesis and cell migration. In addition, the oncogenic roles of WDR1 in tumour cell proliferation and invasion are also characterised. It has been reported that WDR1 is overexpressed in several cancer types, such as breast cancer,^23,24^ ovarian cancer,^33^ glioblastoma^34^ and thyroid cancer.^35^ Specifically, the differential expression of WDR1 in tumour tissues compared with the corresponding non-tumour tissues suggests that WDR1 may act as a therapeutic target.^23,33^ For example, WDR1 can enhance the effects of MRTF-A-induced breast cancer cell migration by promoting the expression of EMT and migration markers through the RhoA-MRTF-A signalling pathway.^23^ In this study, data from our cohort and public database showed that WDR1 was aberrantly overexpressed in PDAC tissues compared with paratumour or normal pancreas tissues. Notably, WDR1 expression increased along with TNM stages. Consistent with previous reports, we showed that WDR1 is crucial for tumour growth and invasive potential in vitro and in vivo by shRNA-mediated loss-of-function studies. Notably, we uncovered a previously unprecedented mechanism of WDR1 in cancers. We showed that WDR1 can modulate the activity of Wnt/β-Catenin signalling by a physical interaction with USP7 and β-Catenin. Given that WDR1 knockdown showed no significant effect on USP7 expression, but reduced the interaction between β-Catenin and USP7 in PDAC cells, we proposed that WDR1 might act as a scaffold to bridges USP7 and β-Catenin, and enhance USP7-mediated β-Catenin deubiquitination. However, the distinct WD domains, culminating in USP7/β-Catenin association, warrant further investigation. Accumulating evidence suggests that USP7 plays crucial roles in cancers, and many advances have been achieved regarding USP7 as a novel therapeutic target for cancer.^36–38^ Of note, targeting USP7-mediated DNMT1 stabilisation in pancreatic cancer holds distinct promise as therapeutic application.^39^ Therefore, blockade of the WDR1–USP7–β-Catenin axis could be a therapeutic strategy for PDAC treatment. Notably, Ji et al. reported that USP7 can act as a negative regulator of Wnt/β-Catenin signalling by promoting Axin deubiquitination and stabilisation during osteoblast and adipocyte differentiation.^40^ Consistent with our findings, two previous studies have demonstrated that USP7 is a positive regulator of Wnt signalling through direct regulation of β-Catenin deubiquitination in colorectal cancer.^38,41^ The differences in cell models used and disease type among these studies may underlie the causes of this contradiction.

Apart from WDR1, several other oncoproteins and tumour suppressors, such as LDHA, CLIC1, ARPC5, ZYX, RCN1, FHIT and CFTR, were identified in this study. In PDAC, LDHA has been reported as a potential biomarker and therapeutic target.^42,43^ Pharmacological inhibition of LDHA by FX11 is able to elicit tumour growth inhibition of pancreatic cancer.^43^ CLIC1 is a newly discovered member of the chloride channel protein family, and has been demonstrated to play a putative oncogene in pancreatic cancer by inhibiting cell proliferation and migration.^44^ FHIT downregulation is an early event in pancreatic cancer.^45^ By regulating gene expression involved in cell-cycle arrest and apoptosis, FHIT may contribute to tumour-suppressor activity.^46^ These studies further support the findings from our screening strategy. However, more studies are warranted to determine whether these proteins are implicated in PDAC development and progression.

In conclusion, we identified an association of the proteome-wide expression profiles with PDAC at different TNM stages, providing a preliminary study of the TNM stage-related proteins in PDAC. A total of 13 differentially expressed proteins in PDAC tissues with different TNM stages were obtained via iTRAQ–2DLC–MS/MS, and the differentially expressed protein WDR1 was selected and verified to further demonstrate its essential roles in the development of PDAC. Our study provides a better understanding of the molecular mechanisms underlying the pathogenesis of PDAC, and presents a promising basis for developing therapeutic targets for the treatment of PDAC.

## Supplementary information

Supplementary information

Supplementary table 3-6

## Data Availability

Data are available upon request.
