# Supplementary material for: WD repeat-containing protein 1 maintains β-Catenin activity to promote pancreatic cancer aggressiveness
Source: Br J Cancer. 2020 Jun 30;123(6):1012–23. doi: 10.1038/s41416-020-0929-0 (PMC7492282; doi:10.1038/s41416-020-0929-0)
Supplement: Supplementary file 1 — Supplementary information [file 41416_2020_929_MOESM1_ESM.docx]

**Supplementary information**


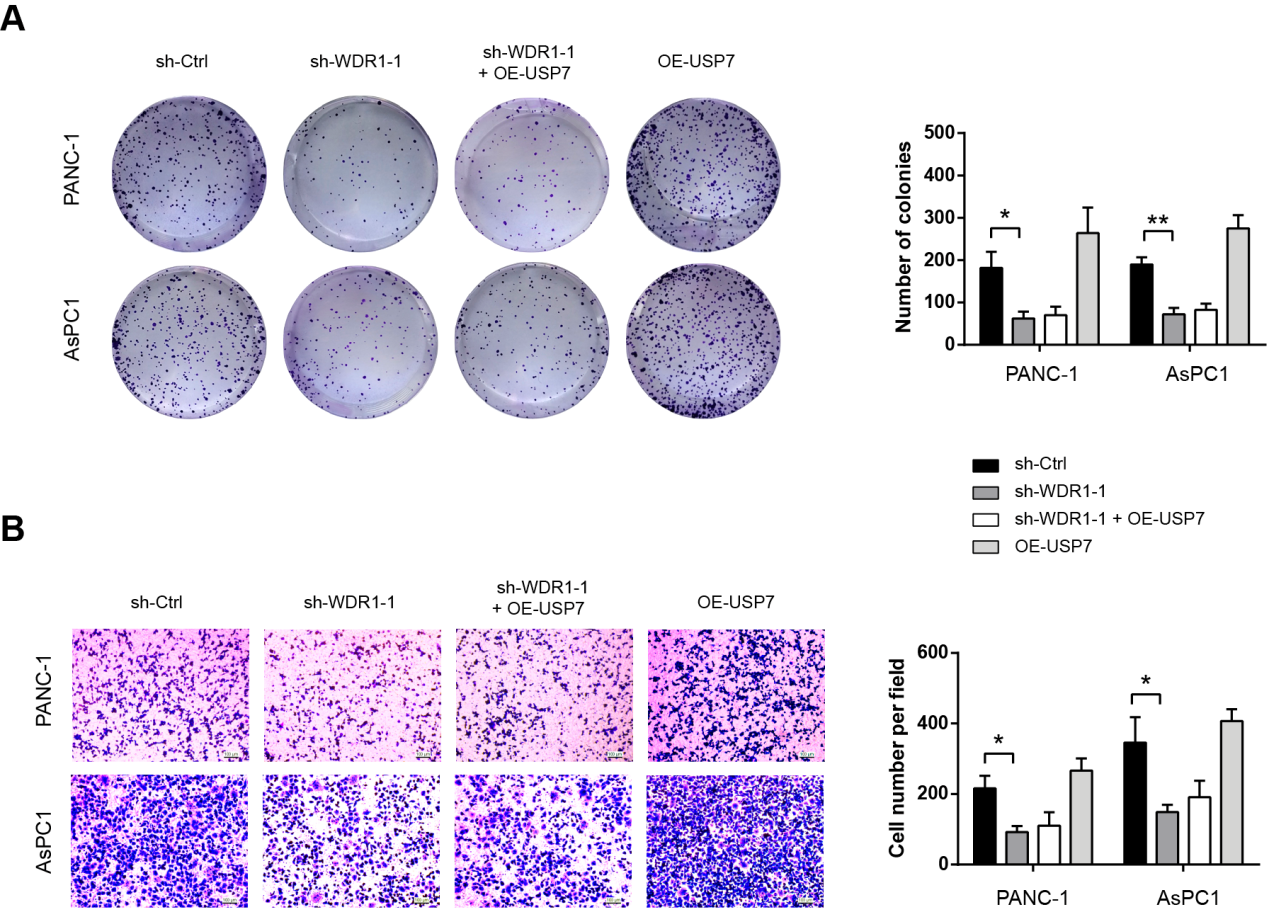


**Supplementary Fig. 1. Ectopic expression of USP7 fails to restore the inhibitory roles of WDR1 on cell proliferation and invasion.** (**A**) The effect of USP7 overexpression on cell proliferation of sh-Ctrl and sh-WDR1-1 PANC1 and AsPC1 cells was determined by plate colony formation assay. (**B**) The effect of USP7 overexpression on cell invasion of sh-Ctrl and sh-WDR1-1 PANC1 and AsPC1 cells was determined by transwell assay. *P < 0.05, **P < 0.01.


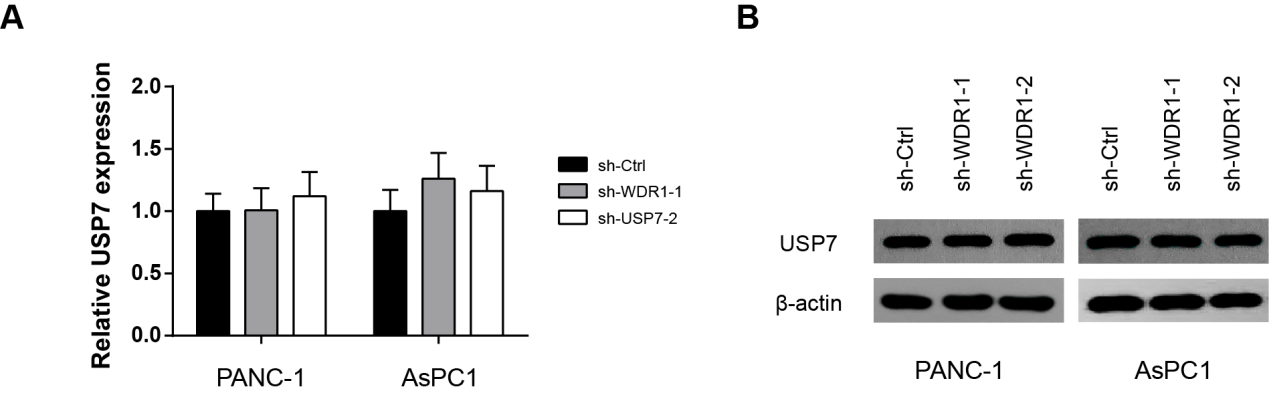


**Supplementary Fig. 2. Effect of WDR1 knockdown on the expression of USP7 in pancreatic cancer.** (**A**) Real-time qPCR analysis of the effect of WDR1 knockdown on the mRNA level of USP7 in PANC1 and AsPC1 cells. (**B**) Western blotting analysis of the effect of WDR1 knockdown on the protein level of USP7 in PANC1 and AsPC1 cells.

| **Supplementary Table S1.Characteristics of study participants (n = 25) used in protein identification** | | | | | |
| --- | --- | --- | --- | --- | --- |
| **Variable** | Group I | Group II | Group III | Group IV | Group V |
|  | Normal | Tumor  (staging I) | Tumor  (staging II) | Tumor  (staging III) | Tumor  (staging IV) |
| **Age** |  |  |  |  |  |
| ≥ 60 years | 0 | 5 | 4 | 3 | 2 |
| < 60 years | 5 | 0 | 1 | 2 | 3 |
| **Gender** |  |  |  |  |  |
| Male | 4 | 1 | 2 | 2 | 2 |
| Female | 1 | 4 | 3 | 3 | 3 |
| **Location** |  |  |  |  |  |
| Head/neck | 5 | 5 | 4 | 3 | 3 |
| Body/tail | 0 | 0 | 1 | 2 | 2 |
| **Neural invasion** |  |  |  |  |  |
| Presence | 0 | 5 | 5 | 5 | 4 |
| Absence | 0 | 0 | 0 | 0 | 1 |
| **Vessel cancer embolus** |  |  |  |  |  |
|  |  |  |  |  |  |
| Presence | 0 | 1 | 1 | 2 | 0 |
| Absence | 0 | 4 | 4 | 3 | 0 |
| **T stage** |  |  |  |  |  |
| T1/T2 | 0 | 0 | 0 | 0 | 1 |
| T3 | 0 | 5 | 5 | 0 | 4 |
| T4 | 0 | 0 | 0 | 5 | 0 |
| **Lymph node metastasis** |  |  |  |  |  |
|  |  |  |  |  |  |
| Presence | 0 | 0 | 5 | 3 | 2 |
| Absence | 0 | 5 | 0 | 2 | 3 |
| **Liver metastasis** |  |  |  |  |  |
| Presence | 0 | 0 | 0 | 0 | 5 |
| Absence | 0 | 0 | 0 | 0 | 0 |
| **TNM stage** |  |  |  |  |  |
| I | 0 | 0 | 0 | 0 | 0 |
| IA/IIA | 0 | 5 | 0 | 0 | 0 |
| IIB | 0 | 0 | 5 | 0 | 0 |
| III | 0 | 0 | 0 | 5 | 0 |
| IV | 0 | 0 | 0 | 0 | 5 |
| **Histologic Grade**  Sample 1  Sample 2  Sample 3  Sample 4  Sample 5 |  | G2  G2  G2  G2  G2-G3 | G2  G2-G3  G2-G3  G2  G2 | G2  G2  G2-G3  G1-G2  G2-G3 | G2-G3  G2  G2  G2  G2-G3 |

**Supplementary Table S2.** Eligibility criteria for selection of the subjects

| 1. Encountered between June 2016 and December 2016  2. Age is not limited  3. Underwent surgical therapy and histologically diagnosed postoperatively by two experienced pathologists  4. In accordance with TNM staging system in PDAC of 7^th^ edition AJCC/UICC Classification of Malignant Tumors  5. Normal pancreatic tissues derived from the donor of liver transplantation without pancreatic disease  6. Tumor tissues of stage IV were confined to those patients who underwent palliative surgery with isolated or single segmental liver metastasis |
| --- |
